# Influence of Transperineal Ultrasound on the POP-Q System in the Surgical Indication of Symptomatic Pelvic Organ Prolapse

**DOI:** 10.3390/jcm13206224

**Published:** 2024-10-18

**Authors:** José Antonio García-Mejido, Ana Hurtado-Guijosa, Alfonso Fernández-Gomez, Fernando Fernández-Palacín, Carolina Lao-Peña, José Antonio Sainz-Bueno

**Affiliations:** 1 Department of Obstetrics and Gynecology, Valme University Hospital, 41014 Seville, Spain; anahurtado96@hotmail.com (A.H.-G.); alfonsofdez4@gmail.com (A.F.-G.); jsainz@us.es (J.A.S.-B.); 2 Department of Surgery, Faculty of Medicine, University of Seville, 41001 Seville, Spain; 3 Department of Statistics and Operational Research, University of Cadiz, 11003 Cadiz, Spain; fernando.fernandez@uca.es; 4 Department of Nursing, Virgen del Rocio University Hospital, 41013 Seville, Spain; carolinalaop@gmail.com

**Keywords:** surgery, pelvic organ prolapse, ultrasonography

## Abstract

**Background/Objectives**: The diagnostic capacity of the preoperative pelvic organ prolapse quantification (POP-Q) system to define surgical pelvic organ prolapse (POP) is sometimes limited. On the other hand, pelvic floor ultrasound can influence the surgical indication for patients with symptomatic POP. Therefore, our objective is to determine how transperineal ultrasound can influence the surgical indication for symptomatic POP. **Methods**: This is a prospective observational study conducted over two years including patients who underwent corrective surgery for symptomatic POP. All patients underwent a preoperative examination using the POP-Q system to assess POP. Patients in whom the pelvic floor specialist had diagnostic doubts about the stage of POP underwent an ultrasound examination of the POP. Before the surgical procedure and with the patient anesthetized, a new clinical examination was performed using the POP-Q system and surgical correction of the POP was executed when the patient had a decline to stage II or higher. Cohen’s kappa coefficient of agreement was used to assess the agreement. **Results**: Of the 180 patients who met the inclusion criteria, 167 were included (99 with preoperative clinical examination and 68 with preoperative clinical examination and ultrasound study). The kappa index for the diagnosis of surgical uterine prolapse of the preoperative clinical examination (moderate correlation) was lower than the ultrasound examination (very good correlation) (0.493 *p* < 0.001 and 0.924 *p* < 0.001). The kappa index for the diagnosis of cervical elongation without surgical uterine prolapse also showed differences between the preoperative clinical examination (good correlation) and the ultrasound examination (very good correlation) (0.749 *p* < 0.001 and 0.853 *p* < 0.001). **Conclusions**: Transperineal ultrasound has a higher concordance than presurgical clinical examination, based on the POP-Q system, for detecting POP with central compartment surgical indication.

## 1. Introduction

Pelvic organ prolapse (POP) is a common disease affecting 25–35% of women [1] and should be considered a health and social problem [2]. The probability of undergoing surgery for POP has been estimated at 11% [3] and POP surgeries are expected to increase by almost 50% by 2050 [4]. For patients with multicompartmental POP, surgical failure rates are up to 58% [5]. Therefore, it is important to understand the causal mechanisms and optimize corrective surgical techniques in order to reduce POP recurrence [5].

The pelvic organ prolapse quantification (POP-Q) system has become the standard staging system for POP [6], establishing itself as the most widely used system in the medical literature [7,8] to assess POP. The interobserver and intraobserver reliability [9] of the POP-Q system has been described, and it is an objective and specific system to describe and stage POP [10]. Although the intraobserver and interobserver reliability of this methodology is good for the anterior and posterior compartment, it is poorer for the central compartment [9]. In addition, with the POP-Q system it has been shown that there are changes in the staging of POP in the preoperative classification with respect to the examination performed in the operating room [11]. It has been determined that POP is more pronounced in the operating room for both the middle and posterior compartments [11]. These differences in the classification of POP severity according to the time of the examination will directly influence surgical planning. In summary, the surgical indication for POP currently depends on the patient’s symptoms and the clinical examination based on the POP-Q system. This system classifies the degree of POP differently depending on the time at which the examination is performed (preoperatively or in the operating room), influencing the planning of the different surgical techniques for patients with POP.

On the other hand, transperineal ultrasound has emerged as a complementary test that can help in the diagnosis of symptomatic POP. The diagnostic capacity of transperineal ultrasound for POP has been shown to have a sensitivity of 60% to 93% and a specificity of 64% to 95% [12,13,14]. Transperineal ultrasound has a number of advantages over the POP-Q system, as it uses a fixed reference point (posterior–inferior border of the pubic symphysis) [12,13,14,15] as opposed to the mobile point used by the POP-Q system (hymen) [10]. In addition, ultrasound allows for the control of confounding factors that are not studied by the POP-Q system, and which may influence the diagnosis of POP, such as the assessment of bladder volume [15], the determination of the contraction of the levator muscle during Valsalva [16] or the duration of Valsalva. Possibly, the most important advantage of transperineal ultrasound compared to the POP-Q system is the better diagnostic capacity for surgical central compartment prolapse [12].

Based on these aspects described in the literature, we consider that pelvic floor ultrasound can influence the surgical indication of symptomatic POP, avoiding the possible diagnostic errors presented by the POP-Q system applied presurgically versus the examination performed in the operating room. Therefore, our objective is to determine how transperineal ultrasound can influence the surgical indication of symptomatic POP.

## 2. Materials and Method

This was a prospective observational study that analyzed for two years (from 1 January 2022 to 31 December 2023) all patients who underwent corrective surgery for symptomatic POP at Valme University Hospital (Seville).

All patients were recruited consecutively from a specialized pelvic floor dysfunction consultation. The patients included had to have a POP with surgical correction criteria (symptomatic POP with a stage 2 or higher) established by a pelvic floor dysfunction specialist. Patients with a history of previous corrective pelvic floor surgery were excluded. The clinical parameters studied were as follows: age, menopausal status, body mass index (BMC), history of childbirth, cesarean section or abortion, and presence and stage of POP (cystocele, uterine prolapse, cervical elongation without uterine prolapse, rectocele and enterocele).

All participants gave their written informed consent to participate in the study. The project was approved, with code 1259-N-20, by the Clinical Research Ethics Committee of the Hospital Universitario Ntra. Sra. de Valme on 24 November 2020.

### 2.1. Preoperative Clinical Examination

The preoperative examination of the patients was performed in a specialized pelvic floor dysfunction consultation by a gynecology specialist specialized in pelvic floor dysfunction, who made the indication for surgical correction of POP. All patients underwent a standardized interview and a clinical examination using the POP-Q system to evaluate POP [10]. Stage 1 was classified when the most distal portion of the prolapse was 1 cm above the hymen, stage 2 was classified when the most distal portion of the prolapse was ±1 cm from the hymen, stage 3 was classified when the descent was +1 cm from the hymen and the protrusion was not greater than 2 cm of the vaginal length and stage 4 was classified when we were facing a complete prolapse.

Surgery was indicated in women with symptomatic POP (stage 2 or greater) who refused or had not had success with non-surgical treatment. In cases where the pelvic floor specialist had diagnostic doubts about the stage of POP (he was not able to determine the exact stage of POP with the clinical examination), a pelvic floor ultrasound was performed.

### 2.2. Ultrasound Examination

Ultrasound examination of the POP was performed only in patients in whom the pelvic floor specialist had diagnostic doubts about the stage of the POP. For the ultrasound study, a Toshiba^®^ 700 Aplio (Toshiba Medical Systems Corp., Tokyo, Japan) was used with a three-dimensional abdominal probe PVT-675 MV covered by a sterile glove. All ultrasounds were performed by an expert gynecologist (with more than 10 years of experience), who was blinded to the clinical examination. Transperineal ultrasound of the pelvic floor was performed following the systematics established in the literature, with the patient in dorsal lithotomy and with the bladder empty [17,18]. The transducer was placed carefully on the patient’s perineum, applying the minimum possible pressure. Captures were performed at rest and in Valsalva (at least 6 s [19]), previously checking that the patient performed the Valsalva correctly to avoid the elevator coactivation bias. To evaluate POP sonographically, the posteroinferior margin of the pubis was used as a reference point, as previously described in the literature [14].

**Figure 1:**
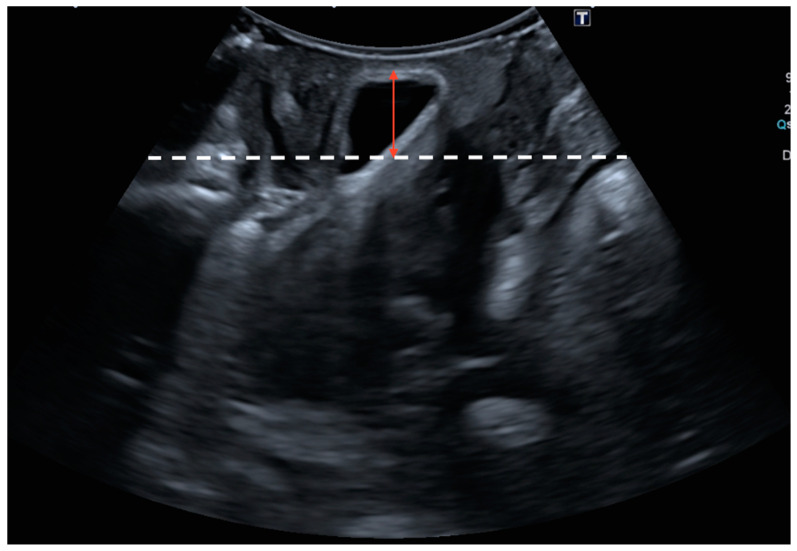
Cystocele was based on a static Valsalva measurement from the posterior–inferior border of the pubic symphysis (white dashed line) to the lowest part of the urinary bladder (red arrow).

**Figure 2:**
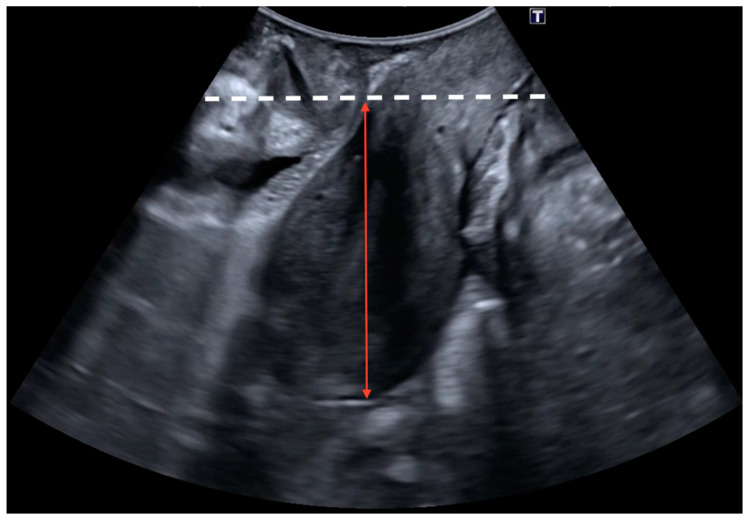
Uterine prolapse was defined as a dynamic measurement (difference between rest and Valsalva) between the posterior–inferior border of the pubic symphysis (white dashed line) and the uterine fundus (red arrow) with a diagnostic cut-off point greater than 15 mm.

**Figure 3:**
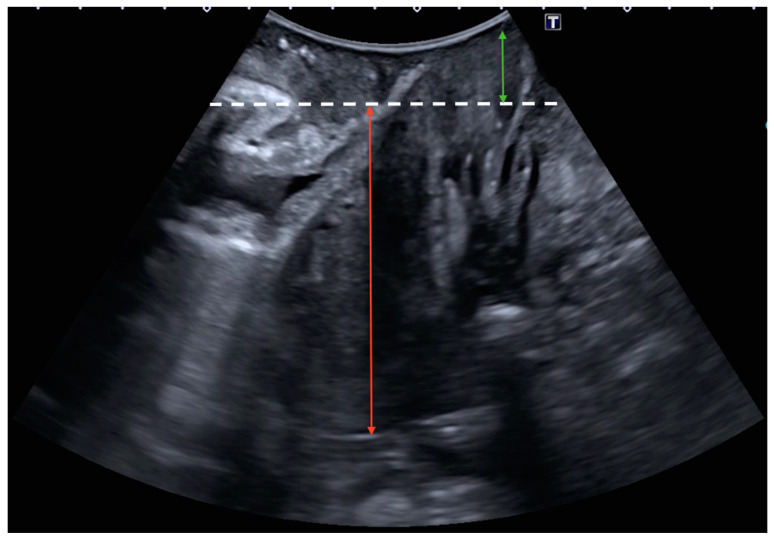
Cervical elongation without uterine prolapse was defined as a dynamic measurement (difference between rest and Valsalva) between the posterior–inferior border of the pubic symphysis (white dashed line) and the uterine fundus (red arrow) with a diagnostic cut-off point of less than 15 mm with a cervical protrusion greater than 15 mm from the posterior–inferior border of the pubic symphysis on Valsalva (green arrow).

**Figure 4:**
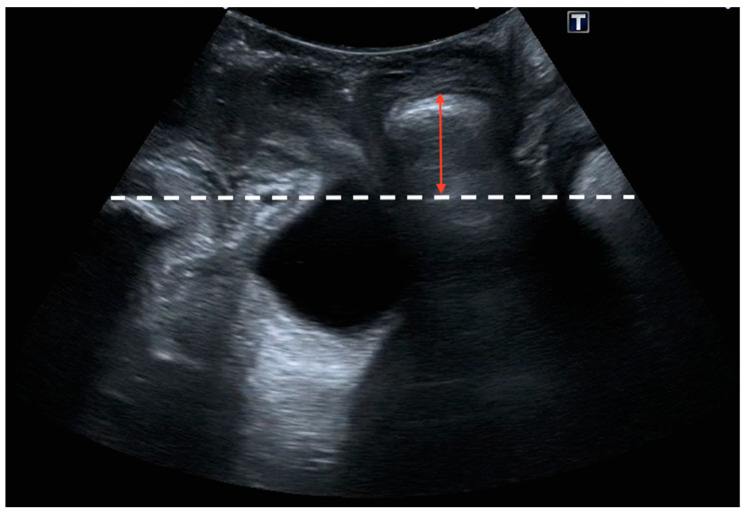
Rectocele was defined as a lowering of the rectum (red arrow) in relation to the posterior–inferior border of the pubic symphysis (white dashed line) with a diagnostic cut-off point greater than 15 mm (static measurement on Valsalva).

The sonographic diagnosis of cystocele was based on a static Valsalva measurement from the posterior–inferior border of the pubic symphysis to the lowest part of the urinary bladder, establishing a diagnostic cut-off point of 10 mm (Figure 1) [13,20]. The sonographic diagnosis of uterine prolapse was defined as a dynamic measurement (difference between rest and Valsalva) between the posterior–inferior border of the pubic symphysis and the uterine fundus with a diagnostic cut-off point greater than 15 mm (Figure 2) [12]. In the case of cervical elongation without uterine prolapse, the diagnosis was based on a dynamic measurement (difference between rest and Valsalva) between the posterior–inferior border of the pubic symphysis and the uterine fundus with a diagnostic cut-off point of less than 15 mm with a cervical protrusion greater than 15 mm from the posterior–inferior border of the pubic symphysis on Valsalva (Figure 3) [12]. The ultrasound diagnosis of rectocele [13,20] and enterocele was defined as a lowering of the rectum or the enterocele region in relation to the posterior–inferior border of the pubic symphysis with a diagnostic cut-off point greater than 15 mm (static measurement on Valsalva) (Figure 4). Rectocele was observed as a herniation of the anterior rectal wall into the vagina and an enterocele was shown as a protrusion of the abdominal contents anterior to the anorectal angle, separating the vagina from the rectal ampulla [21].

### 2.3. Clinical Examination in the Operating Room

Before the surgical procedure and with the patient under anesthesia, the pelvic floor dysfunction specialist, who was blinded to the preoperative examination and the ultrasound examination, performed a new examination using the POP-Q system to evaluate the POP [10]. Finally, this specialist performed a surgical correction of the POP when they presented a descent of stage II or higher.

### 2.4. Postoperative Clinical Examination

A pelvic floor dysfunction examination was performed in the consultation room after surgery by a gynecology specialist specialized in pelvic floor dysfunction, blinded to the procedure and the examinations prior to surgery. The examination was performed using the POP-Q system to assess the recurrence of POP [10] between 60 and 90 days after surgery.

### 2.5. Statistical Analysis

The numerical variables were studied using means and deviations. The Student *t*-test, the Mann–Whitney test or the Shapiro–Wilk test was used to compare numerical variables between the group of patients who did not have an ultrasound evaluation prior to surgery and the group of patients who had an ultrasound evaluation prior to surgery (if they did not meet the hypothesis of normality). The qualitative variables were evaluated as percentages and frequencies and their association was performed using Fisher’s exact test.

The agreement between the presurgical clinical and ultrasound diagnoses of POP was evaluated using Cohen’s kappa coefficient of agreement and its 95% CI, determining the corresponding level of agreement as follows: poor (<0.20), weak (between 0.21 and 0.40), moderate (between 0.41 and 0.60), good (between 0.61 and 0.80) and very good (between 0.81 and 1.00) [22].

## 3. Results

During the study period, a total of 180 patients who met the inclusion criteria were recruited. Thirteen patients were excluded: twelve underwent surgery at other hospitals and one patient did not attend the postoperative check-up. Finally, 167 patients were included, of whom 99 only underwent a preoperative clinical examination using the POP-Q system to evaluate POP and 68 underwent the ultrasound study in addition to the preoperative clinical examination. The general characteristics of both groups together with the preoperative clinical examination using the POP-Q system are reflected in Table 1. Here, we can see that the cases in which the pelvic floor specialist had more diagnostic doubts, and therefore requested the transperineal ultrasound, are those in which the patients are younger (62.7 ± 8.7 vs. 56.8 ± 9.8; *p* < 0.001) and those with cervical elongation without uterine prolapse (9.3% vs. 52.9%; *p* < 0.001). However, it can be seen that the cases in which the pelvic floor specialist had fewer diagnostic doubts, and therefore requested fewer transperineal ultrasounds, are patients with cystoceles (82.8% vs. 63.2%; *p*: 0.006).

Table 2 shows the comparison of postoperative clinical examinations using the POP-Q system between patients with and without ultrasound prior to surgery. The mean time in which the clinical examination was performed in both population groups was 75.7 ± 30.8 and 79.5 ± 34.8 days (*p*: 0.637). No differences were found in POP recurrences between the two population groups at the time of the postoperative examination.

When we observe the kappa index of the patients in the preoperative examination and the ultrasound examination of the POP, using the examination in the operating room of each compartment as the gold standard, we observe different values depending on the type of POP observed (Table 3). We found a very good kappa index for the diagnosis of cystocele both in the preoperative clinical examination (0.815 *p* < 0.001) and in the ultrasound examination (0.811 *p* < 0.001). In the diagnosis of surgical uterine prolapse, the kappa index of the presurgical clinical examination (moderate correlation) was lower than that of the ultrasound examination (very good correlation) (0.493 *p* < 0.001 and 0.924 *p* < 0.001). The presurgical clinical examination also presented a lower kappa index (good correlation) than the ultrasound examination (very good correlation) in the diagnosis of cervical elongation without surgical uterine prolapse (0.749 *p* < 0.001 and 0.853 *p* < 0.001). For the diagnosis of surgical rectocele, both the presurgical clinical examination and the ultrasound examination presented a weak kappa index (0.345 *p* < 0.001 and 0.260 *p* = 0.018). Finally, the presurgical clinical examination presented a lower kappa index (moderate correlation) than the ultrasound examination (good correlation) in the diagnosis of surgical enterocele (0.494 *p* < 0.001 and 0.660 *p* < 0.001).

## 4. Discussion

We observed that in the cases in which the pelvic floor specialist had more diagnostic doubts, and therefore requested more transperineal ultrasounds, they were younger patients (62.7 ± 8.7 vs. 56.8 ± 9.8; *p* < 0.001) and cases of central compartment prolapse. However, in the case of cystoceles, the pelvic floor specialist had fewer diagnostic doubts and requested fewer transperineal ultrasounds (82.8% vs. 63.2%; *p*: 0.006). We also observed that in the presurgical clinical examination of cystoceles there was a very good kappa index (0.815 *p* < 0.001), which was very similar to that presented by the ultrasound examination (0.811 *p* < 0.001), for the diagnosis of surgical cystocele. The good concordance of the position of the urinary bladder between preoperative and operating room evaluations has been previously described [23]. In fact, a later study found no significant differences in the measurements of points Aa and Ba between the preoperative examination and the examination performed in the operating room [11]. Transperineal ultrasound has also shown good diagnostic rates for anterior compartment prolapse with sensitivities ranging from 71.4% to 89.9% and specificities ranging from 64.1 to 82% [13,20].

The correlation of transperineal ultrasound with clinical evaluations for the study of POP has been studied [24], with high concordance observed for the central compartment and greater discrepancies for the anterior and posterior compartments [24]. However, the study included patients with any type of POP [24] and not only patients with surgical POP, as in our study. The POP-Q system has shown a good intraobserver and interobserver correlation for the anterior and posterior compartment, and is poorer for the central compartment (intraobserver variability for point C: 0.765, *p*: 0.0001 and for point D: 0.759, *p*: 0.02) (interobserver variability for point C: 0.522, *p*: 0.0003 and for point D: 0.767, *p*: 0.0004) [9]. In the case of transperineal ultrasound, the ultrasound study of the POP of the central compartment presents an excellent interobserver correlation [25]. In addition, the sensitivity and specificity of transperineal ultrasound for diagnosing central compartment POP are 60.2–81.2% and 64–91.7%, respectively [12,15,20]. When comparing ultrasound with clinical findings in patients with surgical POP, we observed that ultrasound had a better diagnostic capacity in the central compartment [12]. This aspect represents an important advance, since the POP-Q system assessment tends to underestimate central compartment POP in the presurgical examination compared to the examination performed in the operating room [11], with a low concordance between these two examinations being observed for this compartment [11]. Our results are in accordance with those previously described, where we observed that the diagnostic concordance of ultrasound for surgical uterine prolapse and cervical elongation without surgical uterine prolapse are superior to presurgical clinical examination.

In the posterior compartment, especially in surgical rectocele, we observed that both the preoperative clinical examination and the ultrasound examination presented a weak kappa index. In the literature, the diagnostic capacity of ultrasound for rectocele is the most variable among all POP diagnoses, presenting sensitivities and specificities of 70.8–93.1% and 47.1–78%, respectively [13,20]. The increase in the descent of the posterior compartment during the examination in the operating room compared to the preoperative examination has already been described previously [11]. Although we do not know the clear etiology by which this occurs, we consider that it may be influenced by the effect of regional anesthesia since it relaxes the pelvic floor muscles and exerts less counterforce to protrusion [26].

### Strengths and Limitations

The main strength of our work is that we recruited patients from our usual clinical practice for two years; therefore, our results can be reproduced. In addition, we considered that it is the pelvic floor specialist who should consider whether the patient needs an ultrasound or not in the face of diagnostic doubts. Based on this aspect, it has been very useful to understand in which cases the pelvic floor specialist had more diagnostic doubts. These cases in turn (central compartment POP) were those that presented a lower kappa index in the presurgical examination. One of our weak points has been that ultrasound has only been performed in cases with a more complex clinical diagnosis. Therefore, we consider that future research should include populations in which both diagnostic techniques are performed simultaneously. Another weak point is the time that passes between the presurgical examinations (clinical and ultrasound) and the surgical procedure. Our surgical waiting times ranged between 4 and 9 months, and we believe that this aspect should be considered in future studies. A weak point that can be criticized is the moment in which we carry out the postsurgical study of the possible recurrence of POP. However, we used this period because it is the moment in which we protocolized the postsurgical assessment, considering that when there is a recurrence in that period of time it is more attributable to an incorrect POP surgery.

## 5. Conclusions

In conclusion, transperineal ultrasound presents a greater concordance than the presurgical clinical examination, based on the POP-Q system, to detect POP with central compartment surgical indication.

## Figures and Tables

**Table 1:** Comparison of general characteristics and preoperative clinical examination using the POP-Q system between patients without and with preoperative ultrasound.

	Without Ultrasound Examination (*n*: 99)	With Ultrasound Examination (*n*: 68)	*p*	IC 95%
Age	62.7 ± 8.7	56.8 ± 9,8	<0.001	3.1; 8.8
Menopausal status	85 (85.9%)	42 (61.8%)	<0.001	−37.1%; −10.4%
Menopause age	50 ± 3.6	50.6 ± 4.4	0.542	−1; 2
BMC	24.4 ± 4.6	27.2 ± 4.5	0.922	−1.4; 1.3
Obstetric history				
Births	2.8 ± 1.3	2.2 ± 0.9	0.017	−1; −0.001
Cesarean sections	0.03 ± 0.2	0.06 ± 0.2	0.368	−0.001; 0.001
Abortions	0.2 ± 0.6	0.4 ± 0.7	0.246	−0.001; 0.001
Presence of cystocele	82 (82.8%)	43 (63.2%)	0.006	−32.9%; −5.8%
Stage of cystocele				
Stage I	3 (3.7%)	1 (2.3%)	0.591	−7.9%; 7.2%
Stage II	10 (12.2%)	8 (18.6%)		−6.8%; 20.6%
Stage III	69 (84.1%)	34 (79.1%)		−20.1%; 9.0%
Presence of uterine prolapse	43 (43.9%)	21 (30.9%)	0.106	−27.2%; 2.0%
Stage of uterine prolapse				
Stage I	14 (32.6%)	6 (28.6%)	0.049	−26.2%; 20.4%
Stage II	3 (7.0%)	6 (28.6%)		1.0%; 42.1%
Stage III	20 (46.5%)	9 (42.9%)		−28.1%; 21.8%
Stage IV	6 (14.0%)	0 (0%)		−24.7%; 2.3%
Presence of cervical elongation without uterine prolapse	9 (9.3%)	36 (52.9%)	<0.001	29.6%; 55.9%
Stage of cervical elongation without uterine prolapse				
Stage I	1 (11.1%)	1 (2.8%)	0.255	−36.8%; 11.0%
Stage II	1 (11.1%)	13 (36.1%)		−8.8%; 46.1%
Stage III	7 (77.8%)	22 (61.1%)		−42.8%; 18.4%
Presence of rectocele	41 (41.4%)	26 (38.2%)	0.749	−17.9%; 11.9%
Stage of rectocele				
Stage I	22 (53.7%)	9 (34.6%)	0.245	−41.0%; 5.4%
Stage II	9 (22.0%)	10 (38.5%)		−6.0%; 38.1%
Stage III	10 (24.4%)	7 (26.9%)		−18.2%; 24.2%
Presence of enterocele	2 (2.0%)	0 (0%)	0.514	−5.9%; 2.8%
Stage of enterocele				
Stage I	0 (0%)	0 (0%)	---	---
Stage II	1 (50.0%)	0 (0%)		---
Stage III	1 (50.0%)	0 (0%)		---

**Table 2:** Comparisons of postoperative clinical examinations using the POP-Q system between patients without and with preoperative ultrasound.

	Without Ultrasound Examination (*n*: 99)	With Ultrasound Examination (*n*: 68)	*p*	IC 95%
Exploration time after surgery (days)	75.7 ± 30.8	79.5 ± 34.8	0.637	−1.0; 2.0
Presence of cystocele	23 (23.2%)	15 (22.1%)	1	−13.8%; 12.0%
Stage of cystocele				
Stage I	17 (73.9%)	10 (66.7%)	0.858	−36.0%; 21.4%
Stage II	4 (17.4%)	4 (26.7%)		−17.3%; 36.2%
Stage III	2 (8.7%)	1 (6.7%)		−20.2%; 19.7%
Presence of uterine prolapse	1 (1.0%)	2 (2.9%)	0.568	−3.2%; 7.8%
Stage of uterine prolapse				
Stage I	1 (%)	2 (100%)	-----	−59.8%; 76.5%
Stage II	0 (%)	0 (%)		−76.5%; 59.8%
Stage III	0 (%)	0 (%)		−76.5%; 59.8%
Stage IV	0 (%)	0 (%)		−76.5%; 59.8%
Presence of cervical elongation without uterine prolapse	3 (3.1%)	0 (0%)	0.270	−7.3%; 2.2%
Stage of cervical elongation without uterine prolapse				
Stage I	3 (100%)	0 (0%)	-----	-----
Stage II	0 (0%)	0 (0%)		-----
Stage III	0 (0%)	0 (0%)		-----
Presence of rectocele	19 (19.2%)	14 (20.6%)	0.845	−10.7%; 14.0%
Stage of rectocele				
Stage I	14 (73.7%)	10 (71.4%)	0.660	−32.5%; 27.1%
Stage II	5 (26.3%)	3 (21.4%)		−32.3%; 25.1%
Stage III	0 (0%)	1 (7.1%)		−10.9%; 26.3%
Presence of enterocele	0 (0%)	2 (2.9%)	0.164	−1.8%; 8.4%
Stage of enterocele				
Stage I	0 (0%)	0 (0%)	-----	-----
Stage II	0 (0%)	2 (100%)		-----
Stage III	0 (0%)	0 (0%)		-----

**Table 3:** The kappa index is compared between patients with a POP grade 2 or higher in the preoperative examination and the ultrasound diagnosis of POP, using corrective surgery for POP in each compartment as the gold standard (influenced by the clinical examination in the operating room).

	Preoperative Clinical Examination (*n*: 167)	*p* Value (McNemar)	Kappa (*p*)	Ultrasound Examination (*n*: 68)	*p* Value (McNemar)	Kappa (*p*)
Corrective surgery for cystocele	117 (70.1%)	0.219	0.815 (<0.001)	40 (58.8%)	0.388	0.811 (<0.001)
Corrective surgery for uterine prolapse	37 (22.3%)	1	0.493 (<0.001)	17 (25.0%)	0.014	0.924 (<0.001)
Corrective surgery for cervical elongation	35 (21.2%)	1	0.749 (<0.001)	30 (44.1%)	0.454	0.853 (<0.001)
Corrective surgery for rectocele	22 (13.2%)	0.057	0.345 (<0.001)	4 (5.9%)	<0.001	0.260 (0.018)
Corrective surgery for enterocele	1 (0.6%)	1	0.494 (<0.001)	1 (1.5%)	1	0.660 (<0.001)

## Data Availability

Dataset available on request from the authors.

## Abbreviations

POP	Pelvic organ prolapse
POP-Q	Pelvic organ prolapse quantification
BMC	Body mass index
